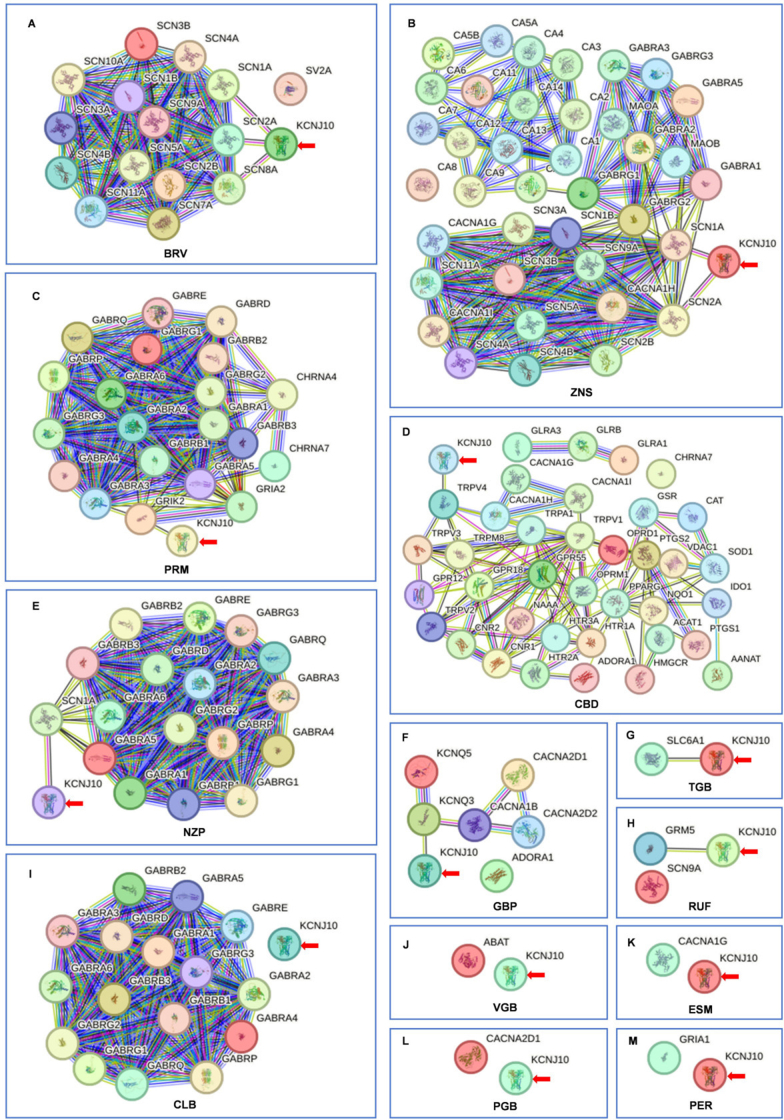# Optimizing anti-seizure medication selection for epilepsy in EAST/SeSAME syndrome: Insights from DrugBank and STRING databases

**DOI:** 10.1016/j.gendis.2025.101852

**Published:** 2025-09-09

**Authors:** Lin-Yan Hu, Lin Wan, Fang Han, Yang Huang, Gang Zhu, Wen-Qi Cao, Wen He, Xiu-Yu Shi, Guang Yang, Li-Ping Zou

**Affiliations:** a Senior Department of Pediatrics, The Seventh Medical Center of Chinese People's Liberation Army General Hospital, Beijing 100007, China; b Department of Pediatrics, The First Medical Centre, Chinese People's Liberation Army General Hospital, Beijing 100853, China

The potassium inwardly rectifying channel subfamily J member 10 (KCNJ10) gene encodes the Kir4.1 inwardly rectifying potassium channel, which is predominantly expressed in the central nervous system, inner ear, and kidneys. Loss-of-function mutations in *KCNJ10* can result in EAST (epilepsy, ataxia, sensorineural deafness, tubulopathy) or SeSAME (seizures, sensorineural deafness, ataxia, mental retardation, and electrolyte imbalance) syndrome.^1^, ^2^, ^3^ The Kir4.1 channels are vital for the brain's functioning, particularly in the cortex, hippocampus, thalamus, and brainstem, where they play a crucial role in regulating astrocyte resting membrane potential, differentiation, and potassium balance. Disruption of these processes can significantly affect cognitive development, motor function, and seizure susceptibility.^4^ In EAST/SeSAME syndrome, seizures are often the earliest and most common symptom to appear in infancy. Broad-spectrum anti-seizure medications (ASMs), such as valproic acid, carbamazepine, oxcarbazepine, lamotrigine, and topiramate, have been shown to be effective.^5^ We present a unique case involving a child with EAST/SeSAME syndrome who has a homozygous missense mutation in *KCNJ10* (c.523C > T, p.Arg175Trp), born to consanguineous parents. The child's seizures began at 48 days old and initially responded well to oxcarbazepine. After a subsequent relapse, topiramate was successful in controlling the seizures, while lacosamide not only failed to prevent another relapse but also aggravated the seizure activity. Considering that *KCNJ10* loss-of-function mutations can compromise Kir4.1 channel activity, ASMs that target sodium channels are theoretically effective for epilepsy associated with this genetic anomaly, as seen in most documented cases. However, the seizure exacerbation observed with lacosamide treatment in this case prompts further investigation into the underlying mechanisms involved.

To deepen our understanding, we carried out an extensive review of reported epilepsy cases linked to *KCNJ10* loss-of-function mutations. Our review encompassed 18 reports, including the case we documented, involving 64 patients with epilepsy (see Table S1). The findings identified 27 pathogenic loss-of-function mutations that resulted in Kir4.1 function reductions ranging from 40% to 100%. The onset of epilepsy occurred between 48 days and 9 months of age. Among the 36 cases with detailed accounts of ASM treatment, the majority responded positively to valproic acid, oxcarbazepine, carbamazepine, lamotrigine, topiramate, phenytoin, and phenobarbital. Notably, two cases (No. 41 and 64 described in Table S1) exhibited no response to lacosamide. Furthermore, eight patients treated with levetiracetam failed to achieve seizure control; however, a switch to other ASMs, such as oxcarbazepine, valproic acid, carbamazepine, or topiramate, led to complete seizure remission in seven of these cases. Our literature review did not uncover a significant link between the age of epilepsy onset, mutation site, retained Kir4.1 function, and the outcomes of epilepsy in EAST/SeSAME syndrome. It was noted, however, that patients consistently showed a lack of response to both levetiracetam and lacosamide.

The lack of response to levetiracetam and lacosamide presents a compelling conundrum. To unravel this, we utilized two databases: DrugBank (version 5.1.12) (https://go.drugbank.com/), which provides comprehensive drug-target information, and STRING (version 12.0) (https://cn.string-db.org/), which offers insights into both established and predicted protein interactions, derived from various sources including genomic context predictions, high-throughput laboratory experiments, conserved co-expression, automated text mining, and established database knowledge. By integrating DrugBank's detailed drug profiles with STRING's protein interaction data, we can delve into the intricate web of drug-target relationships, advancing our understanding of drug mechanisms and potentially guiding the discovery and development of novel therapeutic approaches. We systematically mined DrugBank to identify protein and gene targets associated with ASMs. These targets, along with KCNJ10, were input into STRING's multiple protein search platform to construct an interaction network. The resulting network revealed ASM-mediated molecular hubs that may modulate Kir4.1 function. Our investigation pinpointed nine specific targets through which ASMs interact with the KCNJ10 gene. These targets included sodium voltage-gated channel alpha subunits (SCN1A, SCN2A, and SCN8A), glutamate receptor ionotropic kainate subunits (GRIK1 and GRIK2), metabotropic glutamate receptor 5 (GRM5), transient receptor potential cation channel subfamily V member 4 (TRPV4), sodium- and chloride-dependent GABA transporter 1 (SLC6A1), and potassium voltage-gated channel subfamily KQT member 3 (KCNQ3), as detailed in Table S2. Topiramate engaged with *KCNJ10* through an interaction with both the aforementioned sodium channels (SCN1A, SCN2A, and SCN8A) and kainate receptors (GRIK1 and GRIK2) (Fig. 1A). Lamotrigine, carbamazepine, valproic acid, oxcarbazepine, and phenytoin predominantly affected the sodium channels SCN1A, SCN2A, and SCN8A in their interactions with *KCNJ10* (Fig. 1B–F). Phenobarbital uniquely interacted with KCNJ10 through GRIK2 (Fig. 1G). Levetiracetam engaged with synaptic vesicle glycoprotein 2A (SV2A), ATP binding cassette subfamily B member 1 (ABCB1), and calcium voltage-gated channel subunit alpha 1B (CACNA1B), but did not interact with *KCNJ10* (Fig. 1H). Lacosamide affected SCN3A, SCN9A, SCN10A, SCN11A, and dihydropyrimidinase-like 2 (DPYSL2), yet shows no interaction with *KCNJ10* (Fig. 1I). This difference in target interaction may explain the lack of efficacy of lacosamide in our patient. Extended profiling of 13 additional ASMs revealed that brivaracetam interacted with *KCNJ10* through SCN1A, SCN2A, and SCN8A, while zonisamide did so through SCN1A and SCN2A, primidone through GRIK2, and nitrazepam through SCN1A (Fig. S1A–S1C, S1E). Additionally, cannabidiol interacted with *KCNJ10* via TRPV4, gabapentin via KCNQ3, tiagabine via SLC6A1, and rufinamide via GRM5 (Fig. S1D, S1F–S1H). It is important to note that these last four targets—TRPV4, KCNQ3, SLC6A1, and GRM5—have not yet been clinically validated for the management of EAST/SeSAME syndrome, indicating a need for further research. Conversely, clobazam, vigabatrin, ethosuximide, pregabalin, and perampanel showed no interaction with *KCNJ10* (Fig. S1I–S1M).

**Figure 1:**
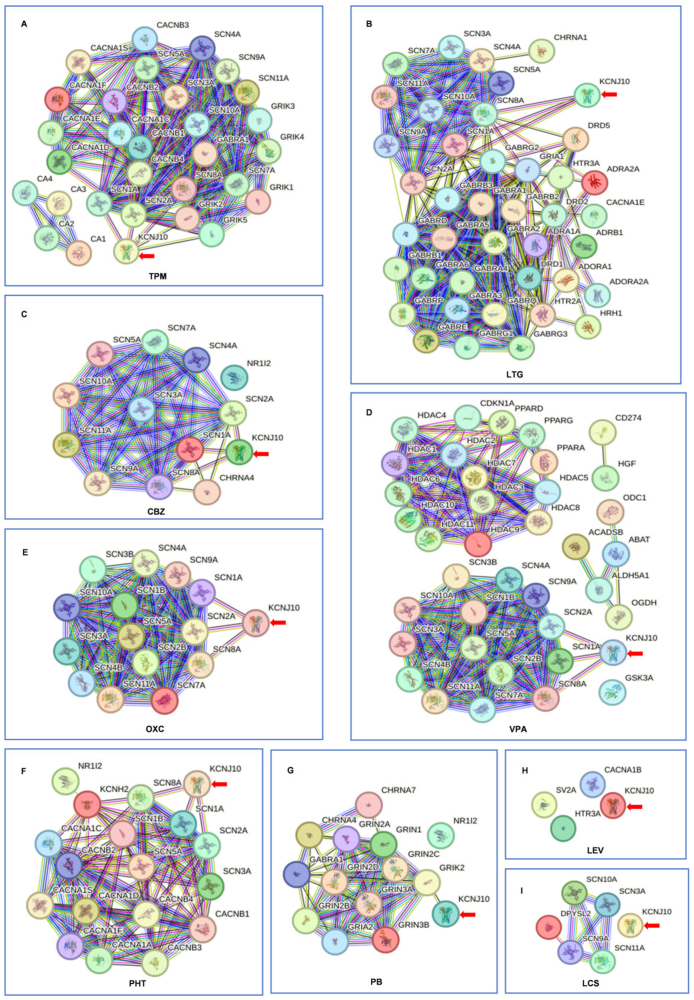
STRING network diagram of the interactions among targets of ASMs and *KCNJ10*. **(A)** TMP-*KCNJ10* interaction is mediated by SCN1A, SCN2A, SCN8A, GRIK1, and GRIK2. TPM exhibits broader target coverage. **(B–F)** LTG, CBZ, VPA, OXC, and PHT engage with *KCNJ10* via SCN1A, SCN2A, and SCN8A. **(G)** PB interacts with *KCNJ10* via GRIK2. **(H)** LEV acts on SV2A, ABCB1, and CACNA1B. No interaction is observed between LEV and *KCNJ10*. **(I)** LCM acts on SCN3A, SCN9A, SCN10A, SCN11A, and DPYSL2. No interaction is observed between LCM and *KCNJ10*. The red arrow annotates the location of *KCNJ10*. The red line indicates the presence of fusion evidence; the green line indicates the presence of neighborhood evidence; the blue line indicates the presence of cooccurrence evidence; the purple line indicates the presence of experimental evidence; the yellow line indicates the presence of textmining evidence; the light blue line indicates the presence of database evidence; and the black line indicates the presence of co-expression evidence. CBZ, carbamazepine; LCM, lacosamide; LEV, levetiracetam; LTG, lamotrigine; OXC, oxcarbazepine; PB, phenobarbital; PHT, phenytoin; TPM, topiramate; VPA, valproate.

Drawing on data from scientific literature, the DrugBank database, and the STRING interaction network, we have identified key insights that inform the strategic selection of ASMs for patients with *KCNJ10* loss-of-function-related epilepsy in EAST/SeSAME syndrome. Our analysis points to topiramate as the most promising option given its interaction with five relevant targets, positioning it as a frontrunner in the treatment of this condition. Additionally, valproic acid, oxcarbazepine, carbamazepine, lamotrigine, phenytoin, and brivaracetam, each exhibiting three interactive sites, are also present as reliable treatment options. Zonisamide, with two points of interaction, along with phenobarbital, primidone, and nitrazepam, each with one, also emerge as viable choices for managing seizures. On the other hand, levetiracetam, lacosamide, clobazam, vigabatrin, ethosuximide, pregabalin, and perampanel are not recommended due to their lack of interaction with *KCNJ10*. In the case study presented, the introduction of lamotrigine, alongside a gradual tapering of lacosamide, successfully reestablished seizure control for the child in question. This practical application underscores the value of our data-driven approach.

It is essential to recognize that although many patients may achieve seizure control with suitable ASMs, there remains a risk of seizure recurrence upon prolonged discontinuation of these drugs, which can occur years later and may escalate to status epilepticus. Consequently, the decision to taper off ASMs should be made with careful consideration and under strict medical supervision.

The insights derived from DrugBank and STRING are instrumental in identifying ASMs that target SCN1A, SCN2A, SCN8A, GRIK1, and GRIK2, which are optimal for managing *KCNJ10* loss-of-function-related epilepsy in EAST/SeSAME syndrome. The efficacy of ASMs that target TRPV4, KCNQ3, SLC6A, and GRM5, however, requires further clinical validation. This knowledge is crucial for the judicious selection of ASMs for patients with EAST/SeSAME syndrome-related epilepsy, ensuring that treatment strategies are personalized and effective within this distinct patient population.

## CRediT authorship contribution statement

**Lin-Yan Hu:** Writing – original draft, Investigation, Funding acquisition, Formal analysis. **Lin Wan:** Writing – original draft, Software, Methodology. **Fang Han:** Writing – original draft, Investigation, Data curation. **Yang Huang:** Writing – original draft, Investigation, Formal analysis. **Gang Zhu:** Writing – original draft, Software. **Wen-Qi Cao:** Writing – original draft, Investigation, Formal analysis. **Wen He:** Writing – original draft, Software, Investigation. **Xiu-Yu Shi:** Writing – review & editing, Supervision, Data curation. **Guang Yang:** Writing – review & editing, Conceptualization. **Li-Ping Zou:** Writing – review & editing, Supervision, Funding acquisition, Conceptualization.

## Ethics declaration

This study was conducted in accordance with the Declaration of Helsinki and approved by the Medical Ethics Committee of the Chinese PLA General Hospital and exempted from ethics board review (Approval No. of Ethics Committee: S2025-129-01). Informed consent was obtained from the parents of the patients.

## Funding

This work was supported by the National Key R&D Program of China (No. 2021YFC2701900; 2023YFC2706300) and the Capital's Funds for Health Improvement and Research (China) (No. 2022-1-5081). None of the funding sources had any involvement in the design, conduct, or interpretation of the study.

## Conflict of interests

None of the authors declare any potential conflicts of interest related to the submission. All authors have made meaningful contributions to the preparation of the manuscript. The corresponding author has full access to all the data in the study and has final responsibility for the decision to submit for publication.

## Figures and Tables

**figs1:**