# Supplementary material for: Optimizing anti-seizure medication selection for epilepsy in EAST/SeSAME syndrome: Insights from DrugBank and STRING databases
Source: Genes Dis. 2025 Sep 9;13(4):101852. doi: 10.1016/j.gendis.2025.101852 (PMC12995480; doi:10.1016/j.gendis.2025.101852)
Supplement: Multimedia component 1 [file mmc1.docx]

**Supplemental data**

Title: Optimizing Anti-Seizure Medication Selection for Epilepsy in EAST/SeSAME Syndrome: Insights from DrugBank and STRING Databases

**Materials and Methods**

**Case presentation**

The detailed information of the course of onset, clinical manifestations, treatment history, investigation results and diagnosis conclusions of a child EAST/SeSAME syndrome were recorded.

**Literature review**

Searches were conducted in MEDLINE and Embase using the terms (1) KCNJ10, (2) EAST syndrome, SeSAME syndrome, or EAST/SeSAME syndrome, and (3) epilepsy. We restricted our search to English-language publications involving human subjects. Duplicates were removed, and the remaining peer-reviewed articles, case reports, case series, and brief communications were included. Data on patients' gender, age at epilepsy onset, gene mutation sites, residual Kir4.1 function, ASM types, and epilepsy outcomes were extracted from the eligible articles.

**ASM-KCNJ10 interaction network mapping**

1.ASMs Drug Target Identification: The targets of ASMs were identified through a systematic search on DrugBank (Version 5.1.12) (https://go.drugbank.com/). The search yielded a list of protein and gene targets of ASM, which are implicated in modulating neuronal signaling and excitability.

2.Protein Interaction Analysis: The STRING database (Version 12.0) (https://cn.string-db.org/) was utilized to investigate potential interactions between the identified ASM targets and the KCNJ10 gene product. The list of ASM targets, along with the KCNJ10 gene, were inputted into STRING's multiple protein search tool to construct an interaction network.

3.Interaction Network Visualization: The interaction network, encompassing both ASM targets and KCNJ10, was generated by STRING. This network was scrutinized for direct and indirect interactions to elucidate the influence of ASMs on Kir4.1 channel function.

4. Functional Enrichment Analysis: STRING's built-in functional enrichment tools were employed to identify significant nodes and interactions within the network. This analysis provided insight into the biological processes and pathways associated with the proteins involved, thereby offering a mechanistic understanding of ASM effects on epileptic seizures in EAST/SeSAME syndrome.

5. Selection of Drug Targets: Proteins that play a central role in the regulation of Kir4.1 channel function or those that demonstrated significant interactions with KCNJ10 were selected as potential drug targets. These targets represent promising avenues for the development of ASMs or for optimizing existing treatment regimens for epilepsy patients with KCNJ10 mutations.

**Results**

**Clinical course and diagnosis of the patients**

A 7-year-old female child presented with spontaneous seizures at 48 days of age, characterized by generalized tonic seizures on a daily basis. Electroencephalo-graph (EEG) demonstrated focal abundant epileptiform discharges, while head MRI scans were unremarkable. Oral OXC treatment resulted in a significant reduction in seizure frequency, along with normalized EEG. By 20 months old, the child experienced aggravated seizures. Then introduction of TPM led to almost 2 years of seizure-free. However, at around 3.5 years old, recurrent seizures reappeared again. EEG revealed abundant, diffuse, asynchronous epileptiform discharges. Adjustments to OXC and TPM dosages resulted in a significant reduction in seizures. Due to intermittent seizures and abnormal EEG findings, LCM was added to the treatment regimen. However, seizures significantly increased with increasing dosages of LCM.

Upon comprehensive reevaluation at our institution, the child was found to have intractable hypokalemia, profound bilateral sensorineural hearing loss, and pervasive developmental delays, including motor and cognitive impairments, as well as ataxia. These clinical manifestations, coupled with persistent hypokalemia, suggested a diagnosis of EAST/SeSAME syndrome. Whole-exome sequencing revealed a homozygous missense mutation in *KCNJ10* (c.523C>T, p.Arg175Trp). Both parents, who are related, were identified as carriers of the heterozygous form of this mutation. Predictive algorithms suggested a high probability that this variant would cause functional disruptions. Based on these findings, the child was formally diagnosed with EAST/SeSAME syndrome.

Supplemental Tables

Table S1. Demographic characteristics of the reported cases with epilepsy related to *KCNJ10* lose-of-function mutation in EAST/SeSAME syndrome.

| Ref | NO. | Sex | Epilepsy Onset age | Mutations | Residual Kir4.1 function | ASMs | The outcome of epilepsy |
| --- | --- | --- | --- | --- | --- | --- | --- |
| 1^[1]^ | 1 | NA | 4 ms | p.C140R(c.418T>C)/  p.C140R (c.418T>C) | 0 | NA | NA |
|  | 2 | F | 3 ms | p.T164I(c.491C>T)/  p.T164I (c.491C>T) | 0 | PB→ PHT | Seizure free with PB and also PHT |
|  | 3 | NA | 3 ms | p.A167V(c.500C>T)/  p.R297C (c.889C>T) | 60% / <10% | NA | NA |
|  | 4 | NA | 3 ms |  |  | NA | NA |
|  | 5 | NA | NA | p.R65P (c.194G>C) /  p.R199Ter (c.595C>T) | <20% / 0 | NA | NA |
| 2 ^[2]^ | 6 | F | 5 ms | p.R65P(c.194G>C)/  p.R65P (c.194G>C) | <20% | None | NA |
|  | 7 | M | 4 ms |  |  | VPA, LTG | Seizure well controlled |
|  | 8 | F | 4 ms |  |  | VPA | Seizure well controlled |
|  | 9 | F | 4 ms |  |  | VPA | Seizure well controlled |
|  | 10 | M | 3 ms | p.G77R (c.229G>C)/  p.G77R (c.229G>C) | <10% | PB | Seizure well controlled |
| 3 ^[3]^ | 11 | M | Infancy | p.R175Q (c.524G>A)/  p.R175Q (c.524G>A) | <20% | NA | NA |
| 4 ^[6]^ | 12 | M | Infancy | p.R65C (c.193G>C)/  p.R65C (c.193G>C) | <20% | NA | NA |
|  | 13 | M | Infancy | p.F75L (c.225T>G)/  p.F75L (c.225T>G) | <10% | NA | NA |
|  | 14 | F | Infancy |  |  | NA | NA |
|  | 15 | M | Infancy | p.R297C (c.889C >T)/  p.R297C (c.889C >T) | <10% | NA | NA |
|  | 16 | M | Infancy | p.V259fsX259 (c.775delG)/  p.V259fsX259 (c.775delG) | 0 | NA | NA |
|  | 17 | F | Infancy |  |  | NA | NA |
| 5 ^[7]^ | 18 | F | 3.5 ms | p.T57I (c.170C>T)/  p.T57I (c.170C>T) | 0 | CBZ, PB→OXC | Seizure free with CBZ and PB from age of 2 to 7, relapsed seizures led to reinitiation of OXC. Prolonged seizure activity at age of 10 years old. |
|  | 19 | F | 6 ms |  |  | PB, DVPX | NA |
|  | 20 | M | 8.5 ms |  |  | PB | NA |
|  | 21 | F | 3 ms |  |  | PB | NA |
| 6 ^[8]^ | 22 | M | NA | p.L68P(203T>C)/  p.I129V(c.385A>G) | NA | NA | NA |
|  | 23 | F | NA |  |  | NA | NA |
| 7 ^[9]^ | 24 | F | 5 ms | p.R65P (c.194G>C)/  p.R65P (c.194G>C) | <20% | DZP, PHE→ CBZ | Seizure free with CBZ |
|  | 25 | M | 4 ms |  |  | PHE→VPA→LTG+VPA | Seizure free with VPA for 3.5 ys, relapsed when VPA was discontinued |
|  | 26 | F | 4 ms |  |  | VPA | Seizure free with VPA |
|  | 27 | F | 4 ms |  |  | VPA | Seizure free with VPA |
|  | 28 | M | 6 ms | p.R65P (c.194G>C) /  p.R199Ter (c.595C>T) | <20% / 0 | CBZ→ LTG | Generalized clonic seizures occur occasionally |
|  | 29 | M | 5 ms | p.R65P  (c.194G>C)/  p.R65P (c.194G>C) | <20% | PB→VPA→VPA+PHE | Seizure free with VPA for 8 ys, relapsed when VPA was discontinued |
|  | 30 | M | 2 ms | p.R297C (c.889C>T)/  p.R297C (c.889C>T) | <10% | PB→ LTG | Seizures were well controlled |
|  | 31 | F | 3 ms | p.F75L (c.225T>G)/  p.F75L (c.225T>G) | <10% | VPA→ LTG | Seizure free with VPA, and relapsed when VPA was discontinued, seizure-free with LTG currently |
|  | 32 | M | 6 ms |  |  | VPA and LEV→LTG | Seizure free with LTG |
| 8 ^[10]^ | 33 | M | 3 ms | p.R204H(c.611G>A)/  p.R204H(c.611G>A) | NA | PB→ VPA | Seizures were significantly reduced after VPA administration |
| 9 ^[11]^ | 34 | NA | NA | p.A167V (c.500C>T)/  p.A167V (c.500C>T) | 60% | NA | NA |
|  | 35 | NA | NA | p.A167V (c.500C>T)/  p.A167V (c.500C>T) | 60% | NA | NA |
|  | 36 | NA | NA | p.F75C (c.224T>G)/  p.F75C (c.224T>G) | 0 | NA | NA |
|  | 37 | NA | NA | p.V91fs197* (c.272delT)/  p.V91fs197* (c.272delT) | 0 | NA | NA |
| 10 ^[12]^ | 38 | M | 3.5 ms | p.R65C(c.193G>C)/  p.F119Gfs*25(c.297_354dup) | <20% / NA | Multiple ASMs → TPM + CBZ | Seizure free with TPM + CBZ |
| 11 ^[13]^ | 39 | F | 3 ms | p.I60T (c.179T>C)/  p.I60T (c.179T>C) | NA | VPA+LTG | Seizure free for 4 years with VPA+LTG, then seizure free for 3 ys without ASMs, seizure relapsed at the age of 7 years old. |
|  | 40 | F | 4 ms |  |  | VPA | Seizure free for 7 ys with VPA, then seizure free for 7 ys without ASMs, seizure relapsed at the age of 14 |
|  | 41 | M | 5 ms |  |  | VPA→LCM | Seizure free with VPA for 3 ys, then seizure free for 11 ys without ASMs, seizure relapsed at the age of 14, no response to LCM |
| 12 ^[14]^ | 42 | M | 3 ms | p.N232Qfs*14(c.693dup)/  p.N232Qfs*14(c.693dup) | NA | PB+ CBZ | Seizures were well controlled, the boy experienced a prolonged seizure at the age of 28 months old, seizure free, epilepsy was well-controlled from late childhood |
|  | 43 | M | 3 ms | p.G275Vfs*7 (c.822delG)/  p.G275Vfs*7 (c.822delG) | NA | VPA+PHB | Seizures was partially controlled, the boy experienced a convulsive status epilepticus at the age of 4 years old. Epilepsy was well-controlled from late childhood |
| 13 ^[15]^ | 44 | F | 7 ms | p.A201T (c.601G > A)/  p.I209T (c.626T > C) | 0 / 37% | LEV→OXC | No response to LEV, seizure free with OXC |
|  | 45 | M | 6 ms |  |  | Allergic to OXC→ LEV→ VPA | No response to LEV, Seizure free with VPA |
| 14 ^[16]^ | 46 | F | 3 ms | p.R65C (c.193G>C)/  p.R65C (c.193G>C) | <20% | VPA→LEV+TPM→ CBZ+LEV→CBZ+VPA | Despite the presence of multiple ASM combinations epilepsy still persists in seizures; CBZ improved psychomotor development, but did not reduce epileptic seizures |
|  | 47 | M | 7ms |  |  | PHB→VPA→ CBZ | Against medical advice, the seizures relapsed within one week of cessation of standard medical therapy |
|  | 48 | F | 4ms |  |  | VPA→ CBZ | CBZ made her calmer and better understanding, but did not relieve her convulsions |
|  | 49 | F | 4ms |  |  | VPA→ CBZ | NA |
| 15 ^[17]^ | 50 | M | 4 ms | p.T57I (c.170C>T)/  p.T57I (c.170C>T) | 0 | VPA→CBZ | Seizure free with VPA for 8ms, VPA was electively changed to CBZ at age of 1 years old, seizure free with CBZ for 2 ys, seizure relapsed when CBZ was tapered. |
|  | 51 | F | 4 ms |  |  | LEV→CBZ | Partial response to LEV, seizure free with CBZ at the age of 9 ms old. |
|  | 52 | F | 3ms |  |  | LEV→TPM→CBZ | No response to LEV, seizure free with TPM, and remained seizure free after switching to CBZ at the age of 3 years old. |
|  | 53 | F | 3ms |  |  | VPA→CBZ | Poor response to VPA, seizure free with CBZ |
|  | 54 | M | 3ms |  |  | LEV→TPM | No response to LEV, seizure free with TPM |
| 16 ^[18]^ | 55 | F | NA | p.T290A( c.A868G)/  p.T290A( c.A868G) | 40% | NA | Seizure free for several years with ASM |
|  | 56 | F | NA |  |  | NA | Seizure free for several years with ASM |
|  | 57 | M | NA |  |  | NA | Seizure free for several years with ASM |
|  | 58 | M | NA |  |  | NA | Seizure free for several years with ASM |
|  | 59 | M | NA |  |  | NA | Seizure free with ASM |
|  | 60 | F | NA |  |  | NA | Seizure free with ASM |
| 17 ^[19]^ | 61 | M | 9ms | p.A167V (c.500C>T)/  p.A167V (c.500C>T) | 60% | VPA | Seizures relapsed after seizure free with VPA for 5 years |
|  | 62 | F | 9ms |  |  | VPA | Seizure free with VPA for 4 years and relapsed because of VPA withdrawal 5 years later |
| 18^[20]^ | 63 | F | 3ms | p.Val186Ala (c.557T >C)  p.Ile129Asn (c.386T >A) | NA | VPA→VPA+LEV→ VPA+LEV+OXC | Poor response to VPA and LEV, seizure free after adding OXC |
| 19 | 64 | F | 48ds | p.A175T(c.523C>T)/  p.A175T (c.523C>T) | NA | OXC→OXC+TPM→OXC+TPM+LCM→ OXC+TPM+LTG | Response to OXC well; Seizure free with the combination of TPM and OXC for almost 2 years; Seizures increased when LCM was added to treat relapsed seizures; Seizures were controlled again after adding LTG and discontinuing LCM. |

Abbreviations: ASMs Anti-seizure medications; CBZ Carbamazepine; LCM Lacoxamide; LEV Levetiracetam; LTG Lamotrigine; OXC Oxcarbazepine; PHB Phenobarbital; PHT Phenytoin; TPM Topiramate; VPA Valproate; EP: epilepsy; M male; F female; NA not available; Y yes; N not; ms months; yrs years; ds days; →Switch to; + Combination

Table S2: The targets of various anti-seizure medications and the targets that interact with the KCNJ10 gene (The last login query time: 11 February 2025).

| ASMs | The targets of the ASM | The targets that interact with the KCNJ10 gene |
| --- | --- | --- |
| Topiramate (TPM) | GABRA1, SCN1A, SCN2A, SCN3A, SCN4A, SCN5A, SCN7A, SCN8A, SCN9A, SCN10A, SCN11A, GRIK1, GRIK2, GRIK3, GRIK4, GRIK5, CA2, CA4, CACNA1C, CACNA1D, CACNA1S, CACNA1F, CACNB1, CACNB2, CACNB3, CACNB4, CA1, CA3, CACNA1E | SCN1A, SCN2A, SCN8A, GRIK2, GRIK5 |
| Lamotrigine (LTG) | CACNA1E, SCN1A, SCN10A, SCN11A, SCN2A, SCN3A, SCN4A, SCN5A, SCN7A, SCN8A, SCN9A, ADORA1, ADORA2A, ADRA1A, ADRA2A, ADRB1, DRD1, DRD5, DRD2, GABRA1, GABRA2, GABRA3, GABRA4, GABRA5, GABRA6, GABRB1, GABRB2, GABRB3, GABRD, GABRE, GABRG1, GABRG2, GABRG3, GABRP, GABRQ, GABRA1, GABRA2, GABRA3, GABRA5, GABRG1, GABRG2, GABRG3, HRH1, OPRK1, CHRNA1, HTR2A, HTR3A, GRIA1 | SCN1A, SCN2A, SCN8A |
| Carbamazepine (CZP) | SCN1A, SCN10A, SCN11A, SCN2A, SCN3A, SCN4A, SCN5A, SCN7A, SCN8A, SCN9A, CHRNA4, NR1I2 | SCN1A, SCN2A, SCN8A |
| Valproate (VPA) | GSK3A, ABAT, ACADSB, OGDH, ALDH5A1, SCN1A, SCN2A, SCN3A, SCN4A, SCN5A, SCN7A, SCN8A, SCN9A, SCN10A, SCN11A, SCN1B, SCN2B, SCN3B, SCN4B, HDAC2, PPARA, PPARD, PPARG, HDAC1, HDAC2, HDAC3, HDAC4, HDAC5, HDAC6, HDAC7, HDAC8, HDAC9, HDAC10, HDAC11, CDKN1A, HGF, ODC1, CD274 | SCN1A, SCN2A, SCN8A |
| Oxcarbazepine (OXC) | SCN1A, SCN10A, SCN11A, SCN2A, SCN3A, SCN4A, SCN5A, SCN7A, SCN8A, SCN9A, SCN1B, SCN2B, SCN3B, SCN4B | SCN1A, SCN2A, SCN8A |
| Phenytoin (PHT) | SCN5A, SCN1A, KCNH2, CACNA1C, CACNA1D, CACNA1F, CACNA1S, CACNB1, CACNB2, CACNB3, CACNB4, CACNA1A, SCN2A, SCN8A, NR1I2, SCN1B, SCN3A | SCN1A, SCN2A, SCN8A |
| Phenobarbital (PB) | GABRA1, CHRNA4, CHRNA7, GRIA2, GRIK2, GRIN1, GRIN2A, GRIN2B, GRIN2C, GRIN2D, GRIN3A, GRIN3B, NR1I2 | GRIK2 |
| Levetiracetam (LEV) | CACNA1B, SV2A, HTR3A | / |
| Lacosamide (LCM) | SCN11A, DPYSL2, SCN9A, SCN3A, SCN10A | / |
| Brivaracetam (BRV) | SV2A, SCN1A, SCN2A, SCN3A, SCN4A, SCN5A, SCN7A, SCN8A, SCN9A, SCN10A, SCN11A, SCN1B, SCN2B, SCN3B, SCN4B | SCN1A, SCN2A, SCN8A |
| Zonisamide (ZNS) | SCN1A, SCN2A, SCN3A, SCN4A, SCN5A, SCN9A, SCN11A, SCN1B, SCN2B, SCN3B, SCN4B, CACNA1G, CACNA1H, CACNA1I, CA1, CA2, CA3, CA4, CA5A, CA5B, CA6, CA7, CA8, CA9, CA10, CA11, CA12, CA13, CA14, MAOB, MAOA, GABRA1, GABRA2, GABRA3, GABRA5, GABRG1, GABRG2, GABRG3 | SCN1A, SCN2A |
| Primidone (PRM) | GABRA2, GABRA3, GABRA4, GABRA5, GABRA6, GABRA1, GABRA2, GABRA3, GABRA4, GABRA5, GABRA6, GABRB1, GABRB2, GABRB3, GABRD, GABRE, GABRG1, GABRG2, GABRG3, GABRP, GABRQ, GABRA1, CHRNA4, CHRNA7, GRIA2, GRIK2 | GRIK2 |
| Cannabidiol (CBD) | CNR1, CNR2, GPR12, GLRA1, GLRB, GLRA3, GPR18, GPR55, HTR1A, HTR2A, CHRNA7, OPRD1, OPRM1, PPARG, TRPV1, CACNA1G, CACNA1H, CACNA1I, TRPA1, TRPM8, TRPV2, TRPV3, TRPV4, VDAC1, HTR3A, ADORA1, PTGS1, PTGS2, ACAT1, HMGCR, GSR, GPX1, IDO1, NQO1, CAT, SOD1, AANAT, NAAA | TRPV4 |
| Nitrazepam (NZP) | GABRA1, GABRA2, GABRA3, GABRA4, GABRA5, GABRA6, GABRB1, GABRB2, GABRB3, GABRD, GABRE, GABRG1, GABRG2, GABRG3, GABRP, GABRQ, GABRA1, GABRA2, GABRA3, GABRA5, GABRG1, GABRG2, GABRG3, SCN1A | SCN1A |
| Gabapentin (GBP) | CACNA2D1, CACNA2D2, CACNA1B, CACNL1A5, ADORA1, KCNQ3, KCNQ5 | KCNQ3 |
| Tiagabine (TGB) | SLC6A1 | SLC6A1 |
| Rufinamide (RUF) | SCN9A, GRM5 | GRM5 |
| clobazam (CLB) | GABRA1, GABRA2, GABRA3, GABRA4, GABRA5, GABRA6, GABRB1, GABRB2, GABRB3, GABRD, GABRE, GABRG1, GABRG2, GABRG3, GABRP, GABRQ, GABRA1, GABRA2, GABRA3, GABRA5, GABRG1, GABRG2, GABRG3 | / |
| Vigabatrin (VGB) | ABAT | / |
| Ethosuximide (ESM) | CACNA1G | / |
| Pregabalin (PGB) | CACNA2D1 | / |
| Perampanel (PER) | GRIA1 | / |

**References:**

1. Scholl UI, Choi M, Liu T, Ramaekers VT, Häusler MG, Grimmer J, Tobe SW, Farhi A, Nelson-Williams C, Lifton RP. Seizures, sensorineural deafness, ataxia, mental retardation, and electrolyte imbalance (SeSAME syndrome) caused by mutations in KCNJ10. Proc Natl Acad Sci USA. 2009; 106(14): 5842-5847. doi: 10.1073/pnas.0901749106.
2. Bockenhauer D, Feather S, Stanescu HC, Bandulik S, Zdebik AA, Reichold M, Tobin J, Lieberer E, Sterner C, Landoure G, Arora R, Sirimanna T, Thompson D, Cross JH, van't Hoff W, Al Masri O, Tullus K, Yeung S, Anikster Y, Klootwijk E, Hubank M, Dillon MJ, Heitzmann D, Arcos-Burgos M, Knepper MA, Dobbie A, Gahl WA, Warth R, Sheridan E, Kleta R. Epilepsy, ataxia, sensorineural deafness, tubulopathy, and KCNJ10 mutations. N Engl J Med. 2009; 360(19):1960-70. doi: 10.1056/NEJMoa0810276.
3. Reichold M, Zdebik AA, Lieberer E, Rapedius M, Schmidt K, Bandulik S, Sterner C, Tegtmeier I, Penton D, Baukrowitz T, Hulton SA, Witzgall R, Ben-Zeev B, Howie AJ, Kleta R, Bockenhauer D, Warth R. KCNJ10 gene mutations causing EAST syndrome (epilepsy, ataxia, sensorineural deafness, and tubulopathy) disrupt channel function. Proc Natl Acad Sci USA. 2010; 107(32): 14490-14495. doi: 10.1073/pnas.1003072107.
4. Sicca F, Ambrosini E, Marchese M, Sforna L, Servettini I, Valvo G, Brignone MS, Lanciotti A, Moro F, Grottesi A, Catacuzzeno L, Baldini S, Hasan S, D'Adamo MC, Franciolini F, Molinari P, Santorelli FM, Pessia M. Gain-of-function defects of astrocytic Kir4.1 channels in children with autism spectrum disorders and epilepsy. Sci Rep. 2016; 6:34325. doi: 10.1038/srep34325.
5. Mir A, Chaudhary M, Alkhaldi H, Alhazmi R, Albaradie R, Housawi Y. Epilepsy in patients with EAST syndrome caused by mutation in the KCNJ10. Brain Dev. 2019; 41(8): 706-715. doi: 10.1016/j.braindev.2019.03.009.
6. Freudenthal B, Kulaveerasingam D, Lingappa L, Shah MA, Brueton L, Wassmer E, Ognjanovic M, Dorison N, Reichold M, Bockenhauer D, Kleta R, Zdebik AA. KCNJ10 mutations disrupt function in patients with EAST syndrome. Nephron Physiol. 2011;119(3): 40-48. doi: 10.1159/000330250.
7. Scholl UI, Dave HB, Lu M, Farhi A, Nelson-Williams C, Listman JA, Lifton RP. SeSAME/EAST syndrome – phenotypic variability and delayed activity of the distal convoluted tubule. PediatrNephrol. 2012; 27(11): 2081-2090. doi: 10.1007/s00467-012-2219-4.
8. Lemke JR, Riesch E, Scheurenbrand T, Schubach M, Wilhelm C, Steiner I, Hansen J, Courage C, Gallati S, Bürki S, Strozzi S, Simonetti BG, Grunt S, Steinlin M, Alber M, Wolff M, Klopstock T, Prott EC, Lorenz R, Spaich C, Rona S, Lakshminarasimhan M, Kröll J, Dorn T, Krämer G, Synofzik M, Becker F, Weber YG, Lerche H, Böhm D, Biskup S. Targeted next generation sequencing as a diagnostic tool in epileptic disorders. Epilepsia. 2012;53(8):1387-98. doi: 10.1111/j.1528-1167.2012.03516.x.
9. Cross JH, Arora R, Heckemann RA, Gunny R, Chong K, Carr L, Baldeweg T, Differ AM, Lench N, Varadkar S, Sirimanna T, Wassmer E, Hulton SA, Ognjanovic M, Ramesh V, Feather S, Kleta R, Hammers A, Bockenhauer D. Neurological features of epilepsy, ataxia, sensorineural deafness, tubulopathy syndrome. Dev Med Child Neurol. 2013;55(9):846-56. doi: 10.1111/dmcn.12171.
10. Kara B, Ekici B, İpekçiB, Aslanger AK, Scholl U. KCNJ10 gene mutation in in an 8-year-old boy with seizures. Acta Neurol Belg. 2013; 113(1): 75-77. doi: 10.1007/s13760-012-0113-2.
11. Parrock S, Hussain S, Issler N, Differ A-M, Lench N, Guarino S, Oosterveld MJS, Keijzer-Veen M, Brilstra E, van Wieringen H, Konijnenberg AY, Amin-Rasip S, Dumitriu S, Klootwijk E, Knoers N, Bockenhauer D, Kleta R, Zdebik AA. KCNJ10 Mutations Display Differential Sensitivity to Heteromerisation with KCNJ16. Nephron Physiol. 2013;123(3-4):7-14. doi: 10.1159/000356353.
12. Papavasiliou A, Foska K, Ioannou J, Nagel M. Epilepsy, ataxia, sensorineural deafness, tubulopathy syndrome in a European child with KCNJ10 mutations: A case report. Sage Open Medical Case Reports. 2017; 5:1-6. doi: 10.1177/2050313X17723549.
13. Muna A. Al Dhaibani, Ayman W. El-Hattab, Kathryn B. Holroyd, Jennifer Orthmann-Murphy, Valerie A. Larson, Khurram A. Siddiqui, Miklos Szolics & Nicoline Schiess: Novel mutation in the KCNJ10 gene in three siblings with seizures, ataxia and no electrolyte abnormalities. Journal of Neurogenetics, 2018; 32(1), 1–5. doi: 10.1080/01677063.2017.1404057
14. Severino M, Lualdi S, Fiorillo C, Striano P, De Toni T, Peluso S, De Michele G,  Rossi A,  Filocamo M,  Bruno C. Unusual white matter involvement in EAST syndrome associated with novel KCNJ10 mutations. J Neurol. 2018; 265: 1419–1425. doi: 10.1007/s00415-018-8826-7.
15. Zhang H, Zhu L, Wang F, Wang R, Hong Y, Chen Y, Zhu B, Gao Y, Luo H, Zhang X, Sun H, Zhou Y, Yao Y and Wang X. Novel KCNJ10 Compound Heterozygous Mutations Causing EAST/SeSAMELike Syndrome Compromise Potassium Channel Function. Front Genet. 2019; 10:912. doi: 10.3389/fgene.2019.00912
16. Celmina M, Micule I, Inashkina I, Audere M, Kuske S, Pereca J, Stavusis J, Pelnena D, Strautmanis J. EAST/SeSAME syndrome: Review of the literature and introduction of four new Latvian patients. Clin Genet. 2019;95(1):63-78. doi: 10.1111/cge.13374.
17. Mir A, Chaudhary M, Alkhaldi H, Alhazmi R, Albaradie R, Housawi Y. Epilepsy in patients with EAST syndrome caused by mutation in the KCNJ10. Brain Dev. 2019; 41(8):706-715. doi: 10.1016/j.braindev.2019.03.009.
18. Nadella RK, Chellappa A, Subramaniam AG, More RP, Shetty S, Prakash S, Ratna N, Vandana VP, Purushottam M, Saini J, Viswanath B, Bindu PS, Nagappa M, Mehta B, Jain S, Kannan R. Identification and functional characterization of two novel mutations in KCNJ10 and PI4KB in SeSAME syndrome without electrolyte imbalance. Hum Genomics. 2019;13(1):53. doi: 10.1186/s40246-019-0236-0.
19. Suzumoto Y, Columbano V, Gervasi L, Giunta R, Mattina T, Trimarchi G, Capolongo G, Simeoni M, Perna AF, Zacchia M, Toriello G, Pollastro RM, Rapisarda F, Capasso G, Trepiccione F. A case series of adult patients affected by EAST/SeSAME syndrome suggests more severe disease in subjects bearing *KCNJ10* truncating mutations. Intractable Rare Dis Res. 2021;10(2):95-101. doi: 10.5582/irdr.2020.03158.
20. Zhang G, Wang M, Chen G, Yang L, Li S, Zhu D. Clinical features and genetic analysis of a child with EAST/SeSAME syndrome. Zhonghua Yi Xue Yi Chuan Xue Za Zhi. 2023; 40(7):838-841. doi: 10.3760/cma.j.cn511374-20220520-00339.

**Supplemental figure legends**

**Figure S1: STRING network diagram illustrates the interactions among target sites of additional ASMs and *KCNJ10*.**

**(A)** BRV connects with *KCNJ10* through SCN1A, SCN2A and SCN8A. **(B, C, E)** ZNS communicates with *KCNJ10* through SCN1A and SCN2A, PRM through GRIK2, NZP through SCN1A. **(D, F, G, H)** CBD links with *KCNJ10* via TRPV4, GBP via KCNQ3, TGB via SLC6A1, and RUF via GRM5. **(I-M)** CLB, VGB, ESM, PGB, and PER showed no interaction with *KCNJ10.*

**Note:** BRV: Brivaracetam, CBD: cannabidiol, CLB: clobazam, ESM: ethosuximide, GBP: gabapentin, NZP: nitrazepam, PER: perampanel, PGB: pregabalin, PRM: primidone, RUF: rufinamide, TGB: tiagabine, VGB: vigabatrin, ZNS: zonisamide. Red line - indicates the presence of fusion evidence; Green line - neighborhood evidence; Blue line - cooccurrence evidence; Purple line - experimental evidence; Yellow line - textmining evidence; Light blue line - database evidence; Black line - coexpression evidence.
